# Supplementary material for: Benefits of FAIMS to Improve the Proteome Coverage of Deteriorated and/or Cross-Linked TMT 10-Plex FFPE Tissue and Plasma-Derived Exosomes Samples
Source: Proteomes. 2023 Oct 24;11(4):35. doi: 10.3390/proteomes11040035 (PMC10661291; doi:10.3390/proteomes11040035)
Supplement: Supplementary file 1 [file proteomes-11-00035-s001.zip › proteomes-2539576-supplementary/Supplementary Figure S2.pptx]

## Slide 1
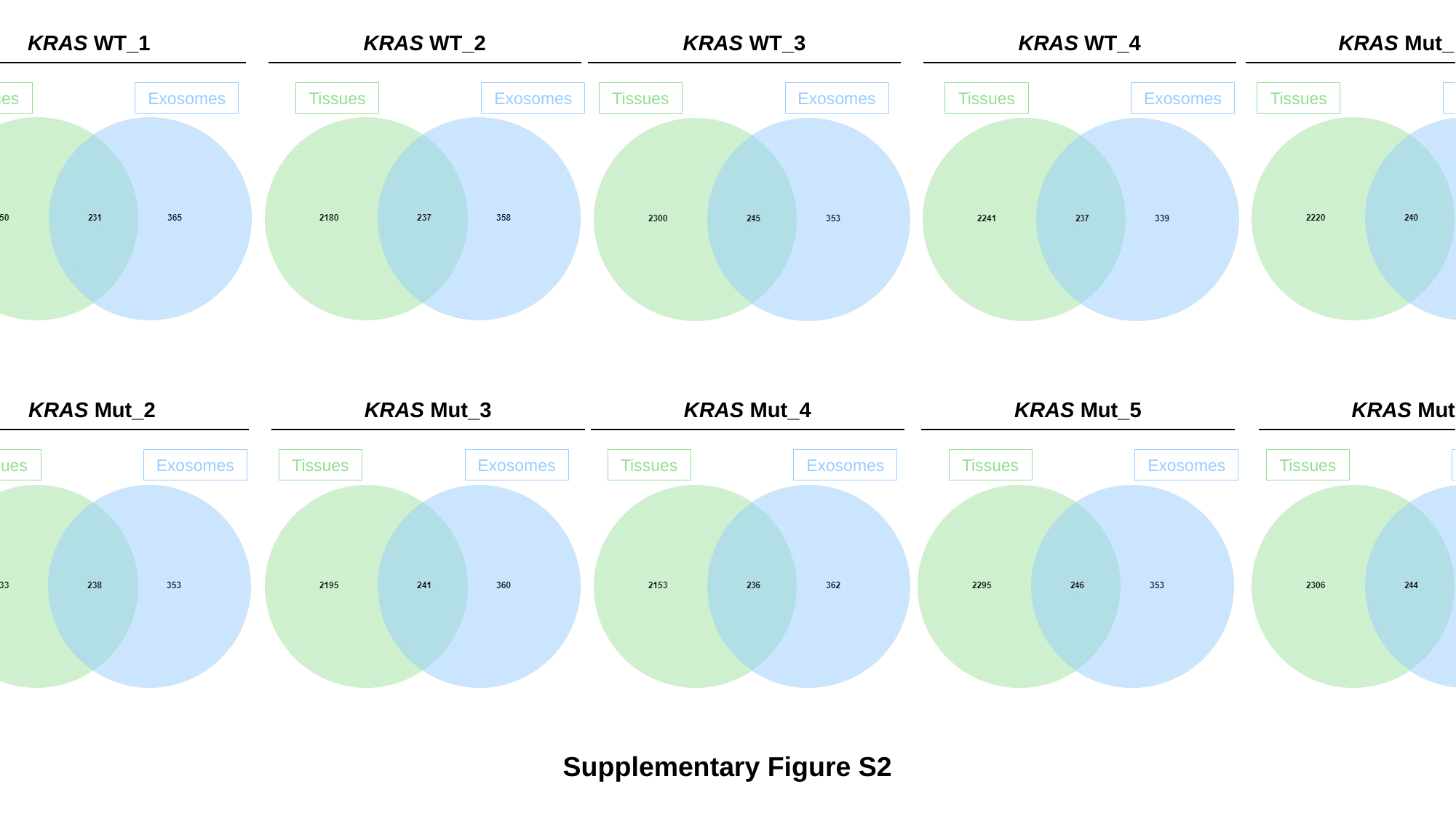

KRAS WT_3
Tissues
Exosomes
KRAS WT_4
Tissues
Exosomes
KRAS Mut_1
Tissues
Exosomes
KRAS WT_1
Tissues
Exosomes
KRAS WT_2
Tissues
Exosomes
KRAS Mut_4
Tissues
Exosomes
KRAS Mut_5
Tissues
Exosomes
KRAS Mut_6
Tissues
Exosomes
KRAS Mut_2
Tissues
Exosomes
KRAS Mut_3
Tissues
Exosomes
Supplementary Figure S2
